# Hepatotoxicity and Drug/Chemical Interaction Toxicity of Nanoclay Particles in Mice

**DOI:** 10.1186/s11671-017-1956-5

**Published:** 2017-03-16

**Authors:** Katsuhiro Isoda, Ryutaro Nagata, Tomoya Hasegawa, Yuichiro Taira, Ikuko Taira, Yoshimi Shimizu, Kazuo Isama, Tetsuji Nishimura, Isao Ishida

**Affiliations:** Faculty of Pharmaceutical Sciences, Teikyo Heisei University, 4-21-2 Nakano-ku, Tokyo, 164-8530 Japan

**Keywords:** Nanoclay particles, Liver injury, Carbon tetrachloride, Paraquat, Cisplatin

## Abstract

Nanomaterials are relatively new and unconventional materials with many useful properties, but their effects on biological systems are poorly understood. Nanoclay is a general term for layered mineral silicate nanoparticles that are ideally suited for use in clay-based nanocomposites. The potential biological hazards of nanoclays have not been addressed, however. Therefore, we investigated the in vivo effects and drug interactions of nanoclays. In mice, administration of nanoclay particles via the tail vein led to acute liver injury. Co-administration of nanoclay and carbon tetrachloride, paraquat, or cisplatin resulted in both liver and kidney injury. Our findings thus indicate that nanoclay particles are potentially hepato- and nephrotoxic.

**Electronic supplementary material:**

The online version of this article (doi:10.1186/s11671-017-1956-5) contains supplementary material, which is available to authorized users.

## Background

Recent advances in technologies for reducing the size of materials have led to the development of innovative nanomaterials with many potential applications. Nanomaterials are frequently used in microelectronics, cosmetics, and sunscreens, and their potential use in drug-delivery systems is being investigated [1–3]. Compared with micromaterials, nanomaterials have various unique physicochemical qualities related to size, surface structure, solubility, and aggregation characteristics. Thus, the reduction in particle size from the micro- to nanoscale could be beneficial in many industrial and scientific applications.

Despite their potential benefits, nanomaterials can exhibit toxicities not associated with micromaterials, making it essential to understand the biological activity and potential toxicity of nanomaterials [4, 5]. Considerable recent research has focused on the human health effects of inhalation of nanomaterials, and a number of risks have been identified. For example, occupational exposure to quartz, mineral dust particles, and asbestos have been shown to induce inflammation, fibrosis, and cytotoxicity in the lungs [6, 7]. Animal studies have revealed that carbon nanotubes are potentially carcinogenic in the lungs [8]. Silica nanoparticles reportedly induce liver injury and can penetrate the blood-brain barrier and enter the brain [9, 10]. Due to differences in the chemical composition of many nanoparticles, their toxicity varies as well [11]. It is possible that chemicals that are otherwise generally nontoxic to humans can be rendered toxic if presented via nano-sized particles.

Due to their viscosity, clay soils are capable of retaining considerable amounts of water. Clays have been used since ancient times as raw materials for ceramics [12], and they are composed of layered silicate minerals such as Si-O_4_, Al-O, and Mg-O arranged in a planar octahedron configuration. The nanoclays used in this study, Si-O_4_ and Al-O, are montmorillonites that form a continuous sheet-like conformation. One side of the sheet-like plane (100 nm or less) of the clay mineral is nanoclay.

Clays are used in a wide variety of products, including paints, cosmetics, and pharmaceuticals [13]. Compared with clays, nanoclays exhibit a number of advantageous properties, including higher workability, mechanical strength, and heat resistance. As such, it is expected that applications for nanoclays in products will increase in the future [14, 15]. However, as clay minerals are generally considered safe to humans, there are no reports examining the potential toxicity of nanoclays. In this study, therefore, the potential toxicity of nanoclay particles was evaluated using acute toxicity testing and drug interaction analyses in mice.

## Methods

### Materials

**Fig. 1:**
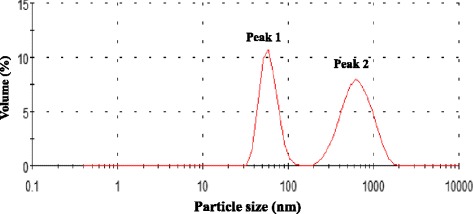
Results of particle diameter measurement of nanoclay particles. The average nanoclay particle diameter of peak 1 was 57.8 ± 12.3 nm, and the average nanoclay particle diameter of peak 2 was 648.3 ± 232.2 nm

Nanoclay particles were obtained from Sigma-Aldrich Co. (St. Louis, MO). The nanoclay particles were in the form of a powder having a layered particle size with one side of 1 nm. For injection, nanoclay particles were dispersed at a concentration of 20 mg/ml in medical water for injection (Fuso Pharmaceutical Industries, Ltd., Japan). The nanoclay particles suspended in the injection water were then stirred and sonicated for 10 min. Analysis of the size distribution of the particles, as determined using a Zetasizer (Sysmex Co., Kobe, Japan) (Fig. 1), revealed an average particle diameter of 57.8 ± 12.3 nm for peak 1 and an average particle diameter of 648.3 ± 232.2 nm for peak 2. In addition, the peak 1 volume was 43.6% and the peak 2 volume was 56.4% (Fig. 1). The suspensions were thoroughly dispersed by sonication before use and then diluted with water. An equal volume of suspension was injected for each treatment. Paraquat and cisplatin were dissolved in saline and stored at −20 °C until use. Carbon tetrachloride was dissolved in olive oil. All reagents used were of research grade. Medical water (Fuso Pharmaceutical Industries, Ltd., Japan), which was used as the solvent for nanoclay particles, was used as the vehicle control.

### Animals

Eight-week-old BALB/c male mice were purchased from Funabashi Farm Co., Ltd. (Chiba, Japan). Animals were maintained in a controlled environment (temperature: 23 ± 1.5 °C; 12-h light/dark cycle) with free access to standard rodent chow and water. The mice were given 1 week to acclimate before the experiments were conducted. The experimental protocols conformed to the ethical guidelines of the Teikyo Heisei University Graduate School of Pharmaceutical Sciences, compiled from the Guidelines for Animal Experimentation of the Japanese Association for Laboratory Animal Sciences.

### Biochemical Analyses

Serum alanine aminotransferase (ALT) and aspartate aminotransferase (AST) are enzymes that leak into the blood during hepatic injury and are important indicators of liver injury [16]. Blood urea nitrogen (BUN) is an index to be inspected when the kidneys are working normally, and increases in BUN values are a measure of deterioration of the kidney function [17]. Serum ALT, AST, and BUN were measured using commercially available kits (Wako Pure Chemical Industries) according to the manufacturer’s protocols. Briefly, collected serum (10 ml) was combined with 1 ml of color A reagent (including urease) and incubated at 37 °C for 15 min. Following the addition of 1 ml of color B reagent, the sample was incubated at 37 °C for 10 min. Absorbance was measured at a wavelength of 570 nm.

### Histologic Analyses

At 24 h after dose administration, animals were sacrificed, and the livers were removed and fixed with 4% paraformaldehyde. Following processing and sectioning, thin tissue sections were stained with hematoxylin and eosin for histologic observation.

### Statistical Analyses

Statistical analyses were performed with Microsoft Excel with the Statcel add-in (EMS Publication Co., Ltd., Saitama, Japan). All data are presented as means ± standard error of the means. The significance of differences between the control and experimental groups was assessed using the Dunnett’s test. A *P* value <0.05 was considered indicative of statistical significance.

## Results and Discussion

**Fig. 2:**
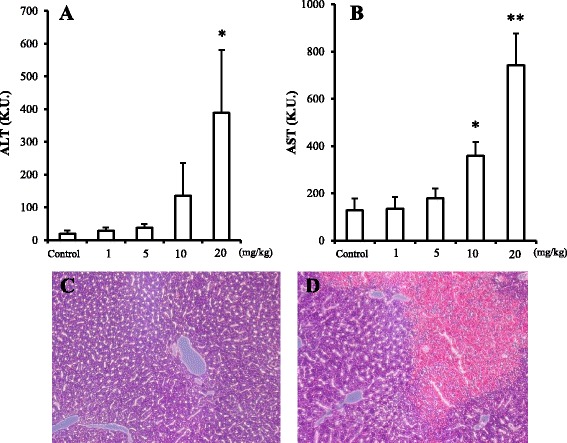
Dose dependence of nanoclay particle-induced liver injury. Serum levels of the liver enzymes alanine aminotransferase (ALT) (**a**) and aspartate aminotransferase (AST) (**b**) were determined using commercially available kits (see Biochemical analyses section) 24 h after intravenous administration of nanoclay particles at the indicated doses. Histologic analyses of tissues of nanoclay particle-treated mice. At 24 h after administration of vehicle (**c**) or nanoclay particles (**d**), tissues were collected, fixed with 4% paraformaldehyde, sectioned, and stained with hematoxylin and eosin. Data are presented as mean ± standard error of the mean (*n* = 4). Significant difference (*P < 0.05, **P < 0.01) compared with the vehicle-treated group

We initially examined the dose dependence of the liver toxicity of nanoclay particles. Acute liver injury was observed at the maximum dose of 20 mg/kg of nanoclay particles (Fig. 2a, b). Hematoxylin and eosin staining of liver tissue indicated extensive injury in mice administered with 20 mg/kg of nanoclay particles (Fig. 2c, d).

**Fig. 3:**
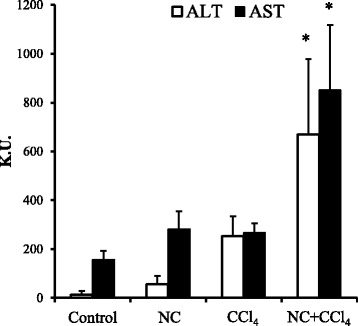
Effect of co-administration of nanoclay particles on carbon tetrachloride-induced toxicity. Mice were injected intraperitoneally with carbon tetrachloride at 0.01 ml/kg together with intravenous injection of vehicle or nanoclay particles (5 mg/kg). At 24 h post-injection, serum levels of the liver enzymes alanine aminotransferase (ALT, *open bars*) and aspartate aminotransferase (AST, *solid bars*) were determined using commercially available kits (see Methods). Data are representative of three independent experiments and are presented as the mean ± standard error of the mean (*n* = 4). Significant difference (*P < 0.05) between vehicle- and carbon tetrachloride-treated groups

We next investigated whether there are interactions between chemicals and nanoclay particles. To prevent interactions between the test chemicals and nanoclay particles prior to administration and absorption, the chemicals were injected intraperitoneally, and the nanoclay particles were injected intravenously. Carbon tetrachloride is known to induce hepatic injury following intraperitoneal administration [18]. We therefore administered carbon tetrachloride to mice at a dose that does not induce hepatic injury (0.01 ml/kg). Treatment with both carbon tetrachloride and nanoclay particles caused significant toxicity, with most of the toxicity associated with the nanoclay particles. Co-administration of carbon tetrachloride and nanoclay particles (5 mg/kg) resulted in increases in serum ALT and AST levels (Fig. 3).

**Fig. 4:**
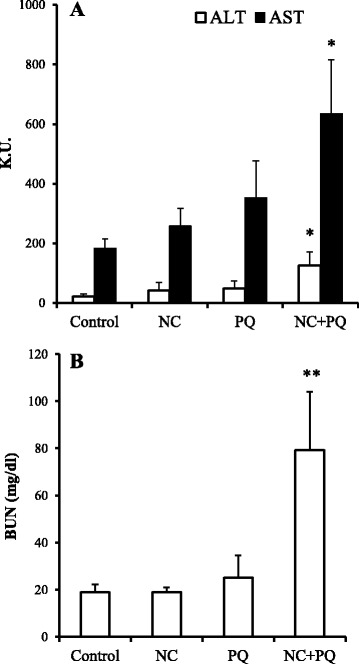
Effect of nanoclay particles (NC) on paraquat (PQ)-induced toxicity. Mice were injected intraperitoneally with PQ at 50 mg/kg together with intravenous injection of vehicle or NC (5 mg/kg). **a** At 24-h post-injection, serum levels of the liver enzymes alanine aminotransferase (ALT, *open bars*) and aspartate aminotransferase (AST, *solid bars*) were determined using commercially available kits (see Methods). **b** Plasma levels of blood urea nitrogen (BUN). Data are representative of three independent experiments and are presented as mean ± standard error of the mean (*n* = 4). Significant difference (*P < 0.05, **P < 0.01) between vehicle- and PQ-treated groups

We also investigated the potential impact of interaction with paraquat on the toxicity of nanoclay particles. Paraquat is one of the most widely used and highly toxic herbicides. Co-administration of paraquat (50 mg/kg) and nanoclay particles (5 mg/kg) led to synergistic significant elevations in serum ALT, and AST levels compared with nanoclay particles and paraquat administered alone (Fig. 4a). Serum BUN levels were also significantly elevated following co-administration of nanoclay particles and paraquat (Fig. 4b). When nanoclay particles and paraquat were administered at the same time, ALT increased from 41.5 in the nanoclay particles alone group and from 48.3 in the paraquat alone group to 126.6. AST increased from 257.6 in the nanoclay particles alone group and 354.6 in the paraquat alone group to 634.1. By simultaneous administration of nanoclay and paraquat, the rise in ALT was synergistic and the rise in AST was additive. It is necessary to examine the mechanism of exacerbation of liver injury by co-administration of nanoclay particles and paraquat.

**Fig. 5:**
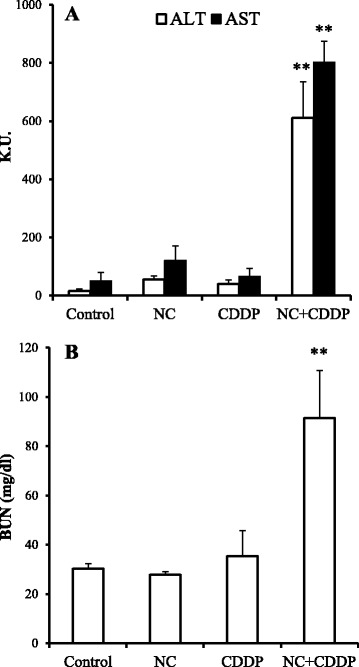
Effect of nanoclay particles (NC) on cisplatin (CDDP)-induced toxicity. Mice were injected intraperitoneally with CDDP at 80 μmol/kg together with intravenous injection of vehicle or NC (5 mg/kg). **a** At 24-h post-injection, serum levels of the liver enzymes alanine aminotransferase (ALT, *open bars*) and aspartate aminotransferase (AST, *solid bars*) were determined using commercially available kits (see Methods). **b** Plasma levels of blood urea nitrogen (BUN). Data are representative of three independent experiments and are presented as the mean ± standard error of the mean (*n* = 4). Significant difference (**P < 0.01) between the vehicle- and CDDP-treated groups

We also investigated the interaction between cisplatin and nanoclay particles. Administration of cisplatin causes a variety of adverse effects, such as renal and hepatic failure [19, 20]. Co-administration of cisplatin (80 μmol/kg) and nanoclay particles (5 mg/kg) produced a synergistic elevation in serum levels of ALT (from 16.3 [control] to 611.7 KU) and AST (from 50.4 [control] to 802.6 KU) (Fig. 5a). Serum BUN levels increased from 30.4 (control) to 90.5 mg/ml (Fig. 5b).

We were able to induce acute liver injury by administering nanoclay particles via the tail vein in mice. Our study is also the first to report liver injury induced by co-administration of nanoclay particles and carbon tetrachloride, paraquat, or cisplatin and kidney injury induced by co-administration of nanoclay particles and paraquat or cisplatin.

Suspension of the montmorillonite nanoclay particles in an aqueous solution resulted in the formation of aggregates with an average particle diameter of 57.8 ± 12.3 nm and 648.3 ± 232.2 nm. However, approximately 46% of the dispersed nanoclay particles exhibited a particle size of ≤100 nm (Fig. 1). The particle size of the nanoclay suspension before sonication was 625 ± 147.6 nm (Additional file 1: Figure S1). Approximately 54% of nanoclay particles suspended by sonication exhibited a size of <100 nm. This result indicates that at least some of the nanoclay particles did not aggregate. We tried to develop a suspension method in which nanoclay particles of 100 nm would be uniformly dispersed, but these efforts were unsuccessful. In the future, it will be necessary to develop a suspension process that does not promote aggregation of the nanoclay particles.

Previously, we reported that hepatic injury was induced by combining silica nanoparticles and polystyrene nanoparticles with carbon tetrachloride or paraquat [9, 21]. We think that it is rare for a nanoclay particles and carbon tetrachloride or paraquat to be used together in real-world situations as they were in this experiment. However, it is possible that hepatic injury may occur by using a liver injury–inducing chemical substance and nanoclay particles in combination. Furthermore, it is necessary to consider combinations of various liver injury-inducing chemicals and nanoclay particles.

Acute liver injury was induced following administration of 10 or 20 mg/kg of nanoclay particles to mice via the caudal vein (Fig. 1). BUN levels, however, did not increase, and no kidney injury was observed (data not shown). In addition, hematoxylin-eosin staining of the lung tissue of mice treated with nanoclay particles provided evidence of lung injury (data not shown). These data suggest that administration of nanoclay particles causes liver injury. Previously, we reported that silica nanoparticles of 70 nm diameter accumulate in the liver, leading to acute liver injury [22]. Yoshida et al. reported that silica nanoparticles are distributed to both the liver and the brain [23]. In addition, Yu et al. reported that silica nanoparticles accumulate in the liver and spleen in mice [24]. Based on these reports, it is reasonable to conclude that the accumulation of nanoclay particles in the liver would also induce liver injury in mice. Future studies should focus on the correlation between liver injury and the amount of nanoclay particles that accumulate in that organ.

We also investigated the combined effects of various chemicals on nanoclay particle-induced toxicity and found that carbon tetrachloride, cisplatin, and paraquat exhibit synergistic toxic effects with nanoclay particles. Liver injury induced by carbon tetrachloride, paraquat, or cisplatin is caused by oxidative stress [25, 26]. Micro- or nano-sized clays also reportedly induce the production of reactive oxygen species (ROS) in vivo [27]. Increased production of ROS following co-administration of nanoclay particles and chemicals is also consistent with liver injury following co-administration. We will perform further biochemical and comprehensive analyses, such as proteomic and genomic studies, to elucidate the mechanism of these synergistic effects.

## Conclusions

In summary, we demonstrated that nanoclay particles can cause liver damage and that this effect can be synergistically exacerbated as a result of interactions with hepatotoxic chemicals or drugs. Further studies based on these data will be required to fully elucidate the toxicologic profiles of nanoparticles proposed for use in human medicine.

## Additional file

Additional file 1: Supplementary Figure 1. (PPTX 49 kb)

## Abbreviations

ALT: Alanine aminotransferase, AST: aspartate aminotransferase
BUN: Blood urea nitrogen
ROS: Reactive oxygen species
