# Supplementary figures and images for: Hepatotoxicity and Drug/Chemical Interaction Toxicity of Nanoclay Particles in Mice
Source: Nanoscale Res Lett. 2017 Mar 16;12:199. doi: 10.1186/s11671-017-1956-5 (PMC5355403; doi:10.1186/s11671-017-1956-5)

## Slide 1
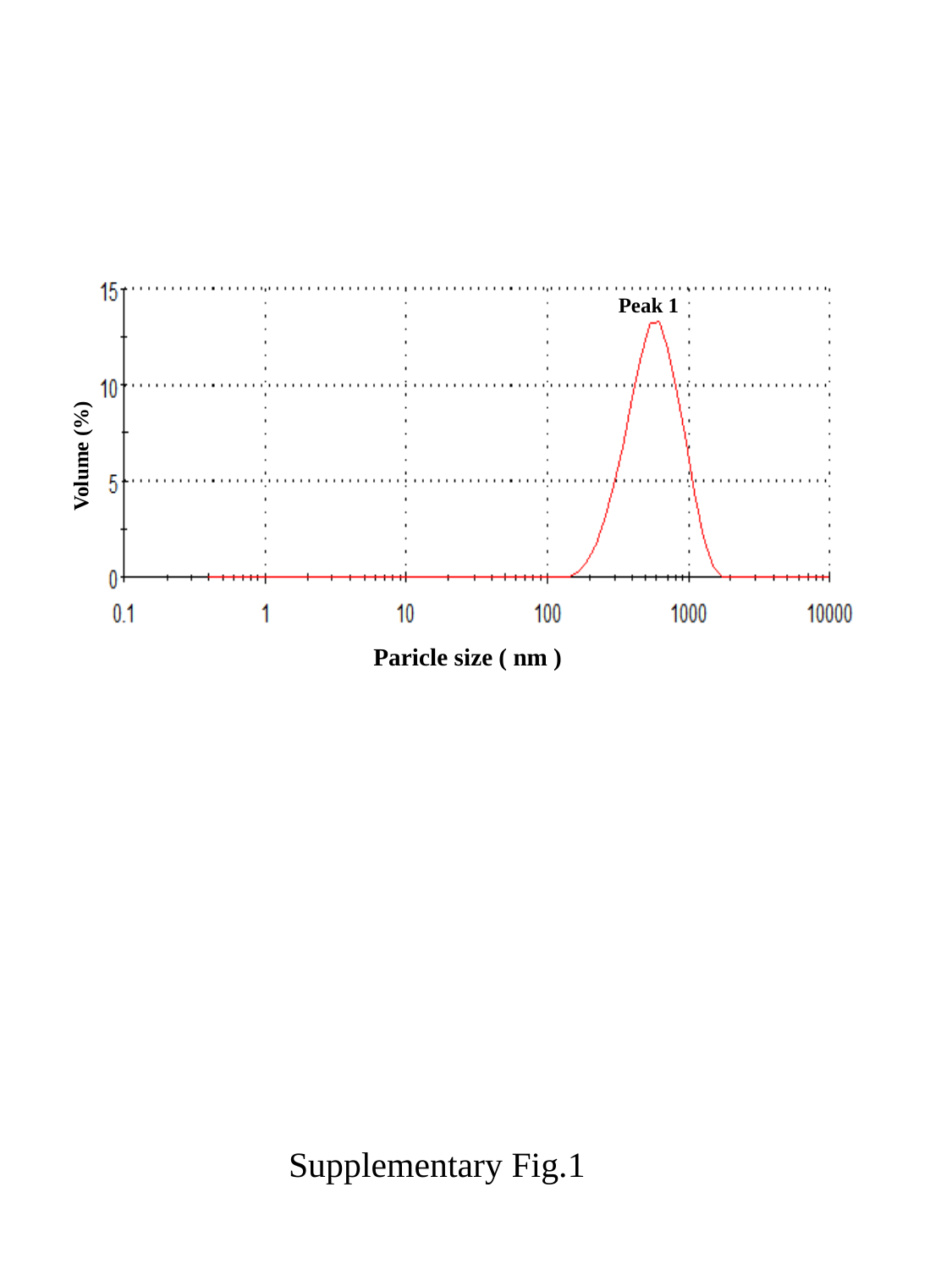

Peak 1
Volume (%)
Paricle size ( nm )
Supplementary Fig.1

Supplement: Additional file 1: — Supplementary Figure 1. (PPTX 49 kb) [file 11671_2017_1956_MOESM1_ESM.pptx]
